# Trends in Frailty Between 1990 and 2020 in Sweden Among 75-, 85-, and 95-Year-Old Women and Men: A Nationwide Study from Sweden

**DOI:** 10.1093/gerona/glac210

**Published:** 2022-10-03

**Authors:** Alexandra M Wennberg, Marcus Ebeling, Stina Ek, Anna Meyer, Mozhu Ding, Mats Talbäck, Karin Modig

**Affiliations:** Unit of Epidemiology, Institute of Environmental Medicine, Karolinska Institutet, Stockholm, Sweden; Unit of Epidemiology, Institute of Environmental Medicine, Karolinska Institutet, Stockholm, Sweden; Unit of Epidemiology, Institute of Environmental Medicine, Karolinska Institutet, Stockholm, Sweden; Unit of Epidemiology, Institute of Environmental Medicine, Karolinska Institutet, Stockholm, Sweden; Unit of Epidemiology, Institute of Environmental Medicine, Karolinska Institutet, Stockholm, Sweden; Unit of Epidemiology, Institute of Environmental Medicine, Karolinska Institutet, Stockholm, Sweden; Unit of Epidemiology, Institute of Environmental Medicine, Karolinska Institutet, Stockholm, Sweden

**Keywords:** Epidemiology, Frailty, Multimorbidities

## Abstract

**Background:**

Aging is the primary risk factor for frailty, which is defined as an inability to respond to acute or chronic stressors. Individuals are living longer with greater multimorbidity, but there is a paucity of evidence examining frailty across birth cohorts and ages.

**Methods:**

We investigated frailty prevalence and its association with mortality at ages 75, 85, and 95 in the 1895–1945 birth cohorts in Sweden with data from population registries. Frailty was assessed with the Hospital Frailty Risk Score (HFRS).

**Results:**

We observed that frailty increased with increasing age and that it has become more common in more recent birth cohorts. At age 75, the percent frail in the Total Population Register increased from 1.1% to 4.6% from birth cohorts 1915–1945, corresponding to calendar years 1990–2020. At age 85, the percentage of frail increased from 3.5% to 11.5% from birth cohorts 1905–1935, and at age 95 from birth cohorts 1895–1925, from 4.7% to 18.7%. Our results show that the increase was primarily driven by an increase in the distribution of individuals with scores in the highest quartile of HFRS, while the bottom 3 quartiles remained relatively stable across birth cohorts. Women accounted for a greater distribution of the overall population and frail population, though these disparities decreased over time. Despite increasing levels of frailty, the relationship between frailty and mortality did not change over time, nor did it differ by sex.

**Conclusion:**

Increased frailty with improved survival points to a chronic condition that could be intervened upon.

The population is aging—since 1980, the older adult population has doubled, and it will double again by 2050, when there will be 2.1 billion people over the age of 60 (1). This increase is partially attributable to improved survival from the disease. However, this has led to an increasing share of individuals who manage chronic conditions over long periods of time (2). On the one hand, health has improved, and diseases have been postponed or prevented. On the other hand, the improved survival might have led to an increasing share of multimorbid or frail individuals in the population.

There are numerous ways to define and measure health in old age (eg, self-reported or objective measures, disease measures or functional abilities), and frailty is one such measure of health. Frailty is a chronic condition affecting functioning and quality of life, and it is the most common disabling condition leading up to death (3). It is defined as the inability to respond to chronic or acute stressors, resulting from a decrease in physical or biological reserve and subsequent inability to maintain homeostasis (4). Frailty affects approximately one-quarter of adults over age 65, but an estimated two-thirds to three-quarters of the oldest old are frail (5,6). Frailty can be conceptualized as either a physical phenotype (ie, weakness, slowness, exhaustion, low physical activity, and unintentional weight loss) or in a cumulative deficit framework, where the accumulation of conditions and diseases define frailty (7). In the cumulative deficit model, multimorbidity and frailty overlap, and frailty scores, such as the Hospital Risk Frailty Score (HFRS), have been developed to count diagnoses to measure frailty (8). This is a regenerating pattern, whereby multimorbidity accelerates disease progression and makes an individual more susceptible to continuing an aging trajectory with an increasing number of chronic conditions and faster decline (9).

It is not clear how frailty has changed over time among older adults in Sweden while life expectancy has increased. Given the trend of improved survival from diseases and an increase in diagnostic capabilities, it might be expected that there has been an increase in the prevalence of frailty over time. However, there has been a simultaneous increase in improved medical care and a greater emphasis on public health initiatives (1,7), perhaps offsetting this expected trend. Overall, there is limited evidence investigating trends in frailty across birth cohorts at the population level. Population-based studies from England and the United States found that more recent cohorts have higher levels of frailty compared to earlier cohorts (10,11). This is echoed in studies investigating trends in multimorbidity over relatively short periods (ie, 9 years), showing increases in prevalence (12). In contrast to this, a study using Swedish data found similar levels of frailty in the 1901–1902 and 1930 birth cohorts at age 70, but frailty was more strongly associated with mortality in the 1901–1902 birth cohort (13). Further understanding frailty and multimorbidity trends, if and how they change over time and at which ages, as well as how the association with mortality changed, is crucial to understand how to care for the aging population. Even if frailty increased, advances in health care might have reduced mortality risks among frail individuals and thus attenuated the association between frailty and mortality.

We aimed to determine how frailty at ages 75, 85, and 95 changed over time and if the association between frailty and mortality has changed between 1990 and 2020 in Sweden. We examined trends in frailty not only by birth cohort and age but also by sex. We assessed frailty with the HFRS, relying on ICD-9 and ICD-10 codes at inpatient and outpatient visits using Swedish population register data.

## Method

### Participants and Data

This study used data from the Swedish National Patient and Population Register, which are linked at the individual level using the Swedish personal identification number assigned to each individual (14). We identified all individuals born 1895–1945, alive and registered in Sweden in 1990 and onwards, and collected their diagnostic codes for the year leading up to their 75th, 85th, and 95th birthdays. The probability of survival from the original birth cohort at ages 75, 85, and 95 is presented in Supplementary Figure 1, for which the aggregated data by Lexis triangles was provided from the Human Mortality Database (15). For analyses examining age-specific mortality trends by frailty status, individuals who migrated during follow-up were excluded, which included 610 individuals at age 75, 470 at age 85, and 376 at age 95. Inpatient and outpatient (from 2001) diagnostic codes, as well as sociodemographic data, including age and sex, were obtained from the population registers (16–18). Notably, the National Patient Registry (NPR) data became available for the entire country starting in 1987, thus, we began our follow-up in 1990 (17) and continued through 2020. Date of death was obtained from the Causes of Death Registry (19). This study was performed in line with the principles of the Declaration of Helsinki. Approval was granted by the Karolinska Institutet Ethics Committee.

### Frailty Assessment

We measured frailty using the Hospital Frailty Risk Score (HFRS), which uses 109 ICD-10 codes which are weighted (0.1–7.1) and summed to create a frailty score (8). Hospitalizations and specialist outpatient care were identified through the linkage of the cohort with the NPR (17) which has coded diagnoses according to ICD-10 since 1997. We additionally translated the codes to ICD-9 to allow for longer follow-up (Supplementary Table 1). Inpatient records were used from 1990 to 2020 and outpatient from 2001 to 2020, duplicate codes on the 3-character level were not used in the calculation of the HFRS. Frailty was assessed using the diagnostic codes from all hospitalizations and outpatient care visits during an entire single year for each subject leading up to their 75th, 85th, and 95th birthday, thus frailty was ascertained on a yearly basis, consistent with previous work (20). For example, for an individual born February 2, 1910, their diagnostic and sociodemographic registry data from February 2, 1994 to February 1, 1995 was used to measure frailty at age 85. If an individual did not seek inpatient or outpatient care for a given year, they were assigned a score of “0” on the HFRS. Individuals were considered frail once their frailty score exceeded 5 (8).

### Statistical Analysis

First, we calculated the HFRS for each birth cohort in the year leading up to their 75th (birth cohorts 1915–1945), 85th (1905–1935), and 95th (1895–1925) birthday and examined trends of frailty by plotting the percentages of people with a score greater than 5, thus considered frail, in bar graphs. We examined these percentages both within the overall population (Total Population Registry) (ie, including those with an assigned value 0) and within the subset of the population that had a specialized care or hospital visit (ie, present in the NPR). Second, we assessed the share of women and men among all individuals considered frail, separately for each birth cohort. To see if the changes in frailty occurred across the whole spectrum of frailty or at one end of the distribution, we additionally analyzed changes in the distribution of HFRS across birth cohorts. HFRS at different quantiles (5%, 25%, 50%, 75%, and 95%) across individuals with non-zero HFRS were calculated separately for each sex and birth cohort. Finally, the distribution of diagnoses among individuals with non-zero HFRS was analyzed by calculating the diagnosis-specific prevalence for each of the 109 ICD-10 hospital codes by birth cohort and age of measurement (75, 85, and 95).

To investigate mortality differences between the frail and the non-frail subgroups, we calculated subgroup-specific death rates by single years of age, sex, and birth cohort groups, and separately for the frail and non-frail subgroups. We grouped 5 consecutive birth cohorts together to increase population size. This allowed us to investigate age-specific mortality differences between the frail and non-frail subgroups across 6 distinct birth cohort groups for each age of frailty measurement. However, the last age for which death rates could be calculated varies by birth cohort group due to the length of follow-up. For frailty measured at age 75, for instance, mortality differences among the most recent birth cohorts (1940–1944) can only be analyzed for age 75, while mortality among the oldest birth cohorts (1915–1919) could theoretically be calculated until age 100. Mortality differences are quantified based on the ratio of the age-specific death rates between the frail and the non-frail subgroups.

Statistical analyses were performed with Stata version 16.1 (StataCorp LLC, College Station, TX) and R version 4.1.1 (R Foundation for Statistical Computing, Vienna, Austria).

## Results

### Time Trends in Prevalence of Frailty

**Figure 1.**
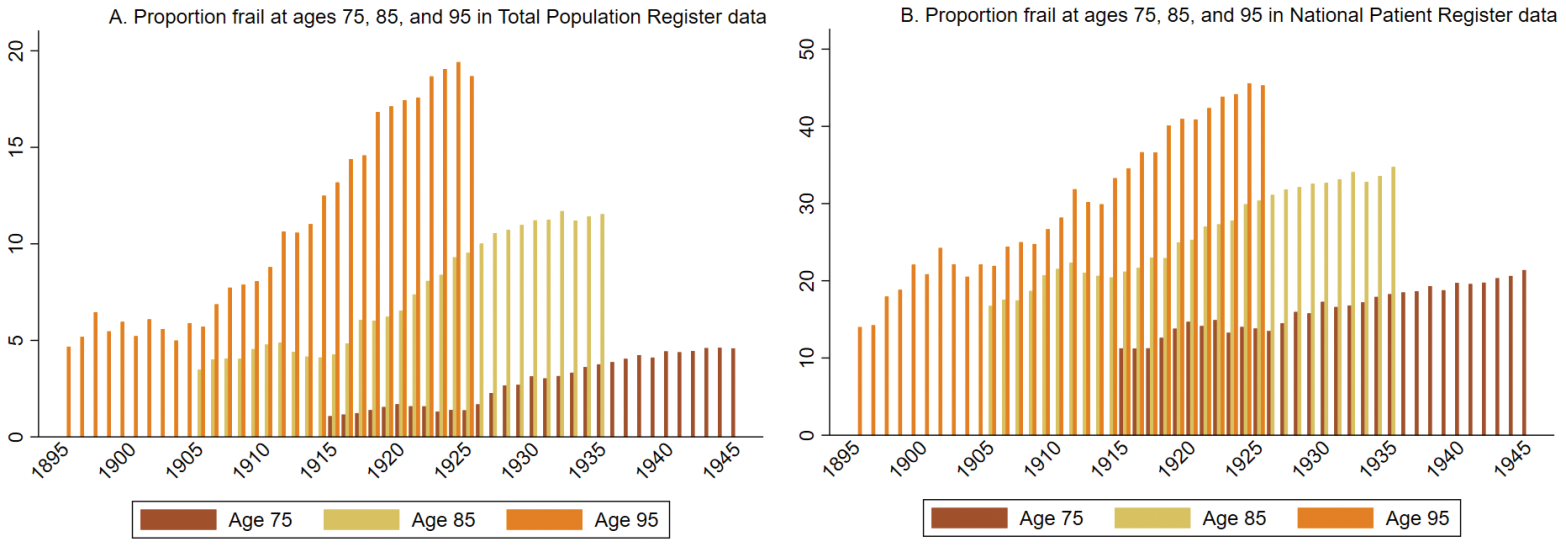
Proportion of frail (Hospital Frailty Risk Score ≥5) individuals in the population (**A**) and in the register (**B**) at ages 75, 85, and 95 in birth cohorts 1895–1945, corresponding to calendar years 1990–2020. Figure 1A represents the proportion of frailty in the total population, including those who were assigned a “0” on the HFRS. Figure 1B represents the proportion of frailty among those who sought inpatient or specialized outpatient care. HFRS = Hospital Frailty Risk Scores.

**Figure 2.**
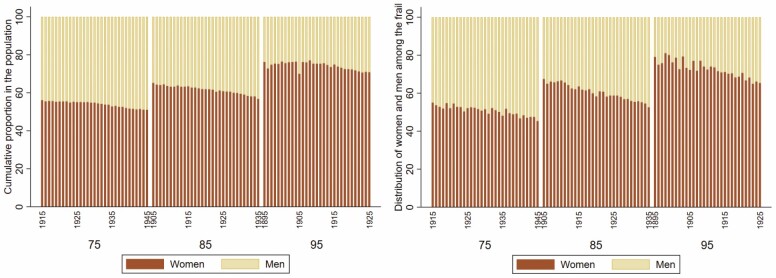
Proportion of women and men among frail and in the total population at ages 75, 85, and 95 from birth cohorts 1895–1945, corresponding to calendar years 1990–2020.

Overall, we observed an increase in frailty score and frailty across birth cohorts. We saw a steady increase in the proportion of frail over time, both in the total population as well as among those hospitalized in the year leading up to the respective ages of 75, 85, and 95 (Figure 1). At age 75, in the total population, frailty increased from 1.1% to 4.6% in birth cohorts 1915 to 1945, and among individuals in the NPR from 11.2% to 21.4% (Figure 1B). Among 85-year olds, frailty increased from 3.5% to 11.5% in the total population, and from 16.8% to 34.7% in the NPR. Among 95-year olds, frailty increased from 4.7% to 18.7% in the total population, and from 14.0% to 45.3% in NPR. There was a small dip at every age corresponding to the late-1990s, likely indicating the change from ICD-9 to the ICD-10. Additionally, because outpatient data only became available in 2001, we conducted a sensitivity analysis excluding diagnoses from the outpatient care register and found the trends were unchanged (results not shown). Of those who were frail, the majority were women; however, the disparity in the distribution of women and men decreased over time (Figure 2), from 55% women to 45% women at age 75; 67% to 53% at age 85; and 81% to 63% at age 95. Women also represented a larger share of the population at ages 75, 85, and 95, but in more recent birth cohorts, the discrepancy between women and men has decreased as more men survived to older ages (Figure 2, Supplementary Figure 1). Overall, the proportion of individuals from each birth cohort that survived until ages 75, 85, and 95 increased over time (Supplementary Figure 1). More specifically, at age 75, cohort survival increased from 62% to 78% among women and from 46% to 70% among men in birth cohorts 1915 to 1945. At age 85, there was an increase in cohort survival from 31% to 51% among women and 15% to 35% among men. At age 95, the cohort survival increased from approximately 4% to 11% among women and 2% to 5% among men.

### Time Trends in Frailty Score Distribution

**Figure 3.**
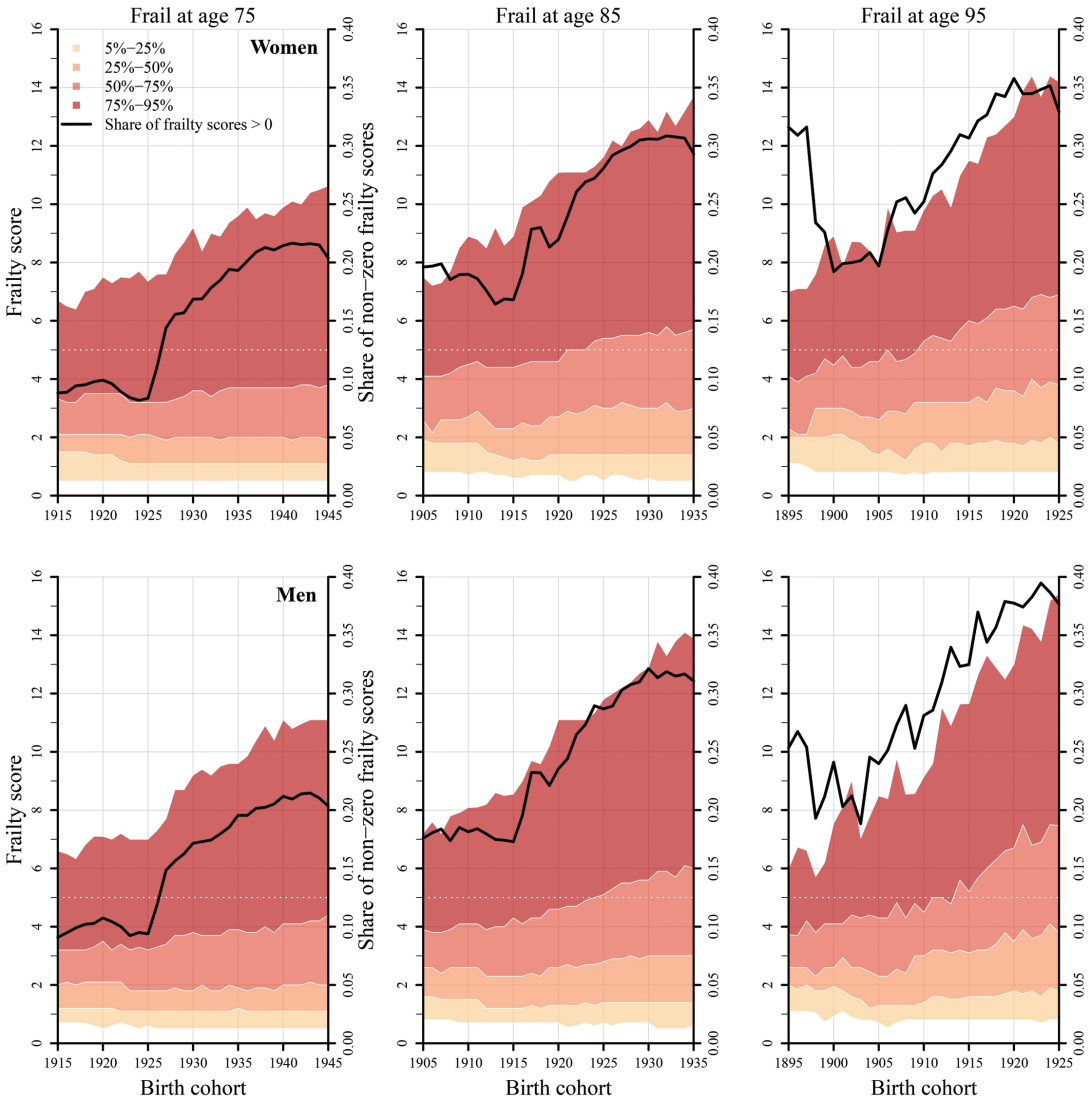
Distribution of Hospital Frailty Risk Scores (HFRS) for women and men over time at ages 75, 85, and 95 in birth cohorts 1895–1945, corresponding to calendar years 1990–2020. The area graphs represent the HFRS (left y-axis) and the proportion of the population with non-zero HFRS (right y-axis) with those scores. The black line represents the share of the population that had a HFRS greater than zero. The dashed line at 5 represents the cut-point used to distinguish frail from non-frail individuals.

The proportion of individuals with frailty scores greater than zero increased over birth cohorts and ages (represented by the solid black line in Figure 3). Notably, there were not substantially different patterns by sex. The increase in the share of non-zero HFRS was accompanied by changes in the distribution of HFRS (Figure 3). Although the median value remained relatively stable at an HFRS of 2 when it was measured at age 75, it increased when measured at older ages. At age 95, the median HFRS increased from approximately 2.5 among those born in 1895 to 4 among those born in 1925. However, the biggest changes were observed for the upper quantiles, particularly among individuals with the highest HRFS. While the 25% of individuals with the highest HFRS at age 85 had a value of 4 or greater among those born in 1905, this value increased to 6 or greater among those born in 1935, and thus, moved the 75% quantile of HFRS above the threshold level of 5. An even stronger increase in the right tail of the HFRS distribution was observed, where, for instance, the upper 5% of individuals with non-zero HFRS at age 85 had a value of 7 or greater in the 1905 birth cohort but a value of almost 14 or greater in the 1935 birth cohort. The value thus doubled over the observation period.

**Figure 4.**
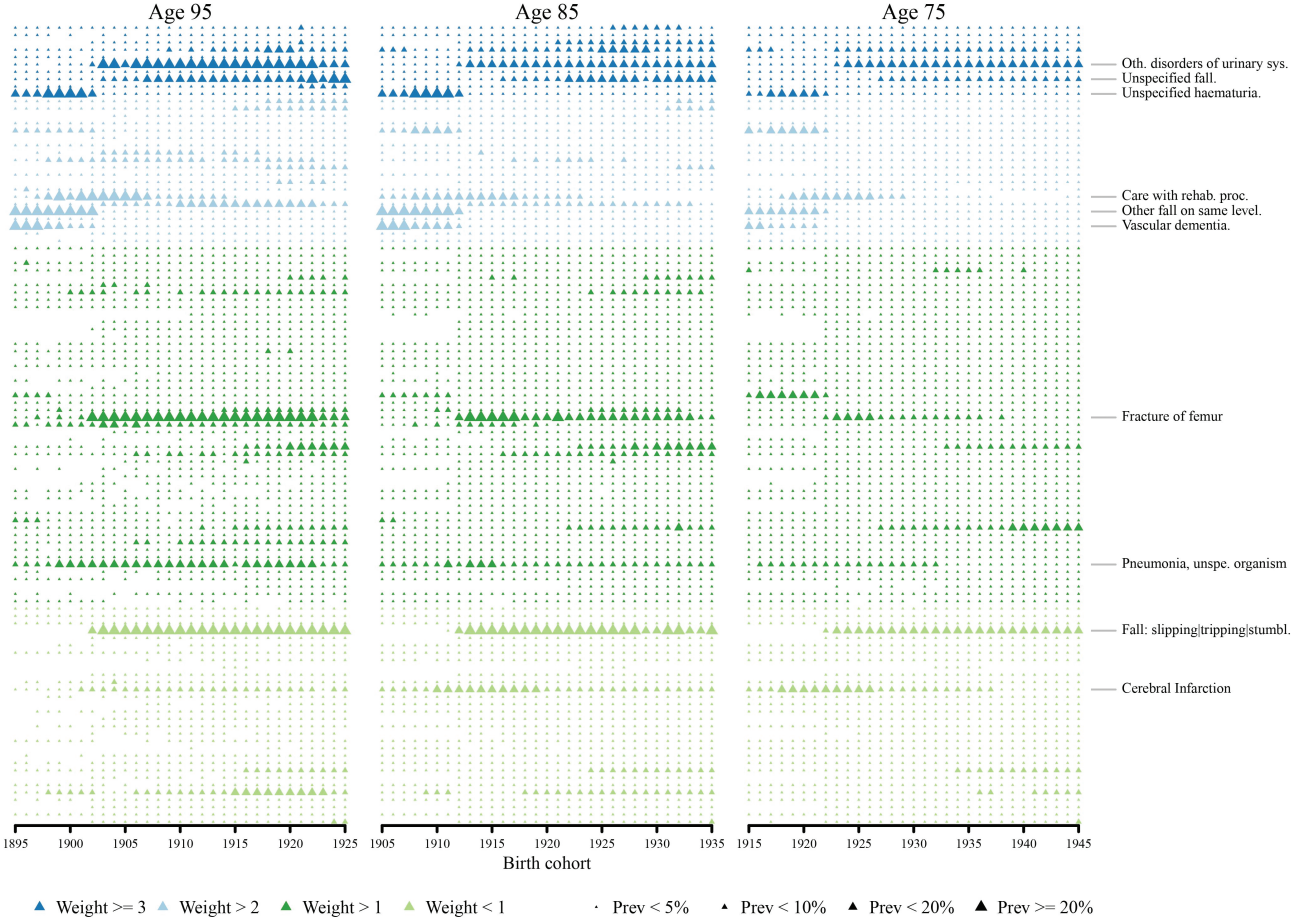
The prevalence of the diagnosis of the conditions in the Hospital Frailty Risk Score (HFRS) at ages 95, 85, and 75 across birth cohorts 1895–1945. The prevalence of the diagnosis (ie, <5%, <10%, <20%, ≥20%) is represented by the size of the triangle and the weight of the condition on the HFRS is represented by the color.

To investigate what could have driven the increase in frailty over time, we additionally explored which diagnoses of the HFRS that were most common at each age over time. In Figure 4, HFRS conditions are presented in order (corresponding to those in Supplementary Table 1), with the prevalence of diagnosis expressed over time at each age. At age 75, vascular dementia, cerebral infarction, and pneumonia became less prevalent over time, while other disorders of the urinary system (including urinary tract infections), chronic renal failure, and falls became more prevalent. Similar patterns were seen at ages 85 and 95, though additionally, fracture of the femur became increasingly prevalent. Across birth cohorts, falls of different types—which appear multiple times on the HFRS—became more commonly diagnosed at each of the 3 ages. Further, among the frail, we found that the mean number of diagnoses an individual has increased with age and over time (Supplementary Figure 2). Although the average increased and there was a pattern for increasing multimorbidity at the 50th and 75th percentiles, those making up the 95th percentile showed the most substantial increase in the number of diagnoses—tripling at age 95 from the 1895 to the 1925 birth cohort.

### Time Trends in the Association Between Frailty and Mortality

**Figure 5.**
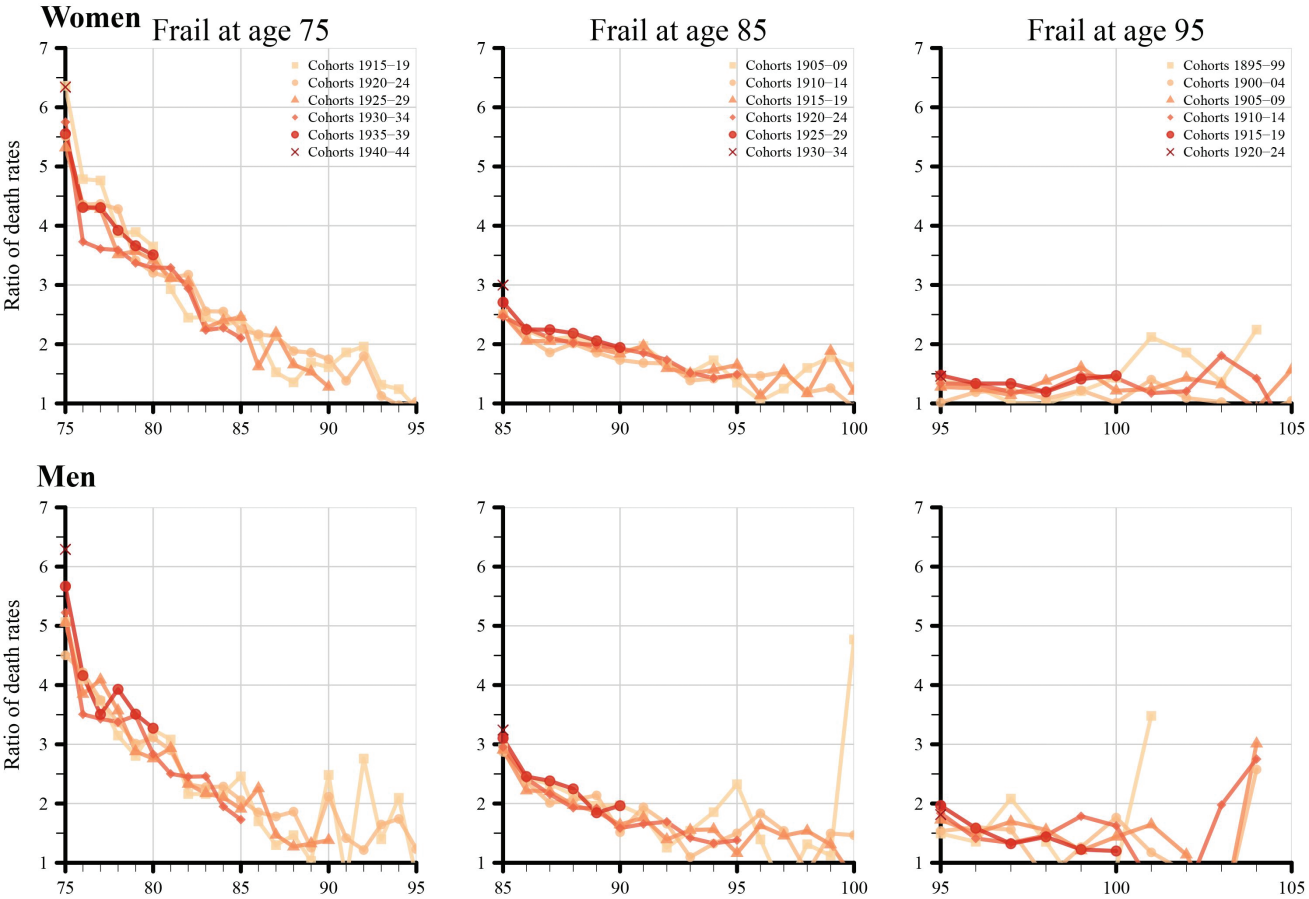
Relative mortality ratios between frail and non-frail stratified by birth cohort (1895–1945) at ages 75, 85, and 95 for women and men. Follow-up ages corresponded to calendar years 1990–2020.

Figure 5 displays the ratio of death rates comparing frail and non-frail individuals in the population over age for different birth cohort groups. We did not observe an increase in the relative difference in mortality between the frail and the non-frail groups across birth cohorts. The ratio between frail and non-frail was largest at age 75 and decreased with increasing age. Notably, we were unable to include the 1945 birth cohort in this figure, because of the lack of follow-up time available. In post hoc analysis, we observed that the rate of mortality improvement in the frail and non-frail groups was comparable over time (results not shown), likely accounting for this lack of increase in death rate despite increasing in frailty.

## Discussion

The present study investigated frailty and associated risk of mortality in Swedish registry data at ages 75, 85, and 95 in the 1895–1945 birth cohorts, corresponding to calendar years 1990–2020. Over this 30-year period, we saw an increase in the share of frail individuals at all ages. While the median HFRS remained relatively stable over time, the 75th percentile moved above the frail non-frail threshold of 5 for frailty at ages 85 and 95. In accordance with the larger number of women in the older population, women also accounted for the majority of the frail population, though the discrepancy between the proportion of frail women and frail men decreased over time. Despite the changes in the distribution of frailty scores, we did not observe a difference in the relationship between frailty and mortality over time. Neither did these patterns or associations differ substantially by sex, although women were more likely to live to older ages. Overall, we saw that the association between frailty and mortality was strongest at age 75 and decreased with increasing age, with a slight increase at age 95 and beyond.

There is little population-level evidence investigating frailty trends and its association with mortality across birth cohorts and ages, and findings from smaller studies are inconclusive. In contrast to our results, another Swedish study that compared a sample from the 1901–1902 (*n* = 965) birth cohort to a sample from the 1930 (*n* = 477) birth cohort found that both cohorts had similar levels of frailty, as measured by the Frailty Index (13). However, a Swedish study of 75-year olds found, like the results presented here, that those born in 1911–1912 (*n* = 591) were less frail than those born in 1930 (*n* = 637) (21). An English study that measured frailty among those aged 50–80+ from 2002 to 2010 also found a trend for higher frailty in recent cohorts compared to earlier cohorts (10). This echoes findings from a U.S. study that examined frailty levels in 88 117 people born from 1914 to 1947 over a 10-year period (11). This study found a quadratic increase in frailty levels over time until the 1942 birth cohort—then those born between 1942 and 1947 showed a more gradual increase in frailty levels over time. Although the present study included birth cohorts from this same period (the first half of the 1940s), we did not observe a similar trend in these later birth cohorts. However, we measured frailty in these birth cohorts at only one age (ie, 75). Neither of these larger studies investigated mortality outcomes, but, in contrast to our findings, both Swedish studies found frailty was more strongly associated with mortality in the earlier birth cohorts (13,21), which may partially be due to methodological differences.

Indeed, it is difficult to directly compare our findings to those from these previous studies, which used the frailty index to assess frailty (10,11,13,21), while here we used the HFRS. The Frailty Index is a self-report measure that considers aspects of well-being, including functionality, mental health, and subjective health. In this study, we used diagnostic codes to assess frailty, thus mirroring more somatic diseases than self-perceived health. Yet the HFRS is not particularly sensitive and may miss some frail individuals at the lower (healthier) end of the frailty spectrum (22,23). However, the studies that showed an increase in frailty severity over time (10,11,13) are consistent with our finding of an increase in the frailty score at quantiles above the median. Though increased diagnostics over time may partially explain increasing frailty prevalence, we found that the level of frailty increased even in recent years, when diagnostic tools have been fairly stable, suggesting diagnostic differences do not completely account for this trend. In general, the evidence points to more people living with frailty, which is likely also a reflection of increased longevity and disease survival compared to earlier birth cohorts. This may seem antithetical to the compression of morbidity hypothesis developed by Fries (24), particularly because we saw frailty prevalence increase even at 75. Yet, mortality rates among the frail in comparison to the non-frail remained constant over time, suggesting the mortality improved at a similar pace. Moreover, the results from the study may support a different interpretation of Fries’ original model. For example, Manton hypothesized that increased longevity would be accompanied by a change in the distribution of disease types, with an increase in the number of years spent with moderate health conditions though fewer years spent with serious health conditions (25). Our finding that although frailty is becoming more common in the population, the association between frailty and mortality has not changed over time suggests that people may be living longer with moderate health conditions, but they are better managed. The same conditions do not lead to death as they used to (26,27). We saw that the stable relationship between frailty and mortality is attributable to equivalent rates of mortality improvement in both the frail and non-frail groups. This is consistent with findings from a recent study in a smaller sample (28), which suggests that mortality in the frail and non-frail subgroups has improved at an approximately similar pace. The relatively stronger association between frailty and mortality at younger ages than older is expected since frailty at younger ages is less common, and the non-frail population has a rather low mortality at these ages.

Looking into which diagnoses within HRFS had become more or less common over time, and at different ages, we saw that at age 75, cerebral infarction, a condition that often leads to death, was diagnosed more often in earlier birth cohorts (29). In more recent birth cohorts, the increase in renal failure diagnosis, which is also associated with mortality, as well as other comorbid conditions, may be contributing to our observation of a stronger association between frailty and mortality at younger ages (30). The pattern we observed showing that chronic renal failure and urinary tract disorders became more common matches findings from other studies showing that these conditions are becoming more prevalent and more serious, with more complications (31,32). This pattern may be indicative of a larger trend in the growing number and complications from recurrent or chronic events and conditions. Notably, events that can lead to disability (eg, falls, femur fracture) were also common at all ages, across all birth cohorts, which is consistent with evidence showing these events are becoming more common with more severe consequences (33).

Although we observed that among the frail, women accounted for a greater proportion, and that women were more likely to live to older ages, we did not see a difference in frailty trends based on HFRS estimates or its relationship with mortality by sex. There is a sex-frailty paradox, such that although women are more likely to live to older ages (34,35), they have greater burden of comorbidities, disability, frailty, and poorer functioning at older ages (36–39). Specifically, older women, as compared to men, are far more likely to experience restrictions in mobility, self-care, and ability to perform household tasks (40). One study among nonagenarians found that men have much higher mortality rates but manage activities of daily living items much better than women do (41). Morbidity development trajectories are also different between men and women. Women show a dramatic linear increase in limiting, chronic illness from their sixties to their eighties whereas men show a nonlinear trajectory that tends to increase just prior to death (42). Moreover, having more comorbidities is more strongly associated with physical dysfunction in women compared to men (43). The findings from our study showing that more women survived to older ages and were more likely to be frail compared to men among the surviving individuals are consistent with the sex paradox presented by others. Similarly, the finding that the difference between women and men in frailty decreased over time is in line with other studies (44).

This study has several strengths, including the population-based data, lack of attrition, and a 30-year follow-up period. However, some limitations must be considered. The use of NPR and diagnostic codes may underestimate frailty among some individuals who do not seek care. Relatedly, the frailty estimates here relied on inpatient and outpatient registered diagnoses and did not account for conditions that may be treated in other settings (eg, primary care, home care). This likely accounts for the discrepancy between our work, showing about 20% of 95-year olds are frail in the population, while most estimates suggest that, among the oldest old, the prevalence of frailty is around two-thirds (5). However, if we restrict our sample to those who were present in the Total Patient Register, that is seeking specialist care or being hospitalized, this proportion is above 40%, which may be a more accurate snapshot. Further, prior to 1987, there was no nation-wide coverage of the NPR, thus limiting our ability to go back in time and to lower ages without losing the possibility to examine the entire population of Sweden. From 2001, outpatient data became available, which meant more care visits were captured from then until the end of follow-up. However, in sensitivity analysis, we found the same trend when using only inpatient data, thus suggesting that perhaps the improved diagnostics over time (2) likely more substantially contribute to the increase in observed frailty.

## Conclusion

In this nationwide study, we observed frailty to be more prevalent in later birth cohorts at all studied ages; however, increases primarily took place in the upper quantiles of the frailty scale. Women constituted a greater share of frail individuals at all studied ages, but over time the gap between women and men decreased—perhaps as a consequence of more men surviving to higher ages. Although frailty increased in the population over time, we did not observe a higher risk of death among the frail, suggesting that mortality improvements occurred at an equal rate in both the frail and non-frail populations. The improved survival also points to a chronic condition that could be intervened upon.

## Supplementary Material

Supplementary data are available at *The Journals of Gerontology, Series A: Biological Sciences and Medical Sciences* online.

Supplementary Table 1. ICD-9 and -10 codes and associated weights summed to determine the Hospital Frailty Risk Score for each individual.

Supplementary Figure 1. Probability of survival from birth to age 75, 85, or 95 from the original birth cohort (1895-1945) to the follow-up year (1990-2020) stratified by sex.

Supplementary Figure 2. The average (black line), median (green line), 75^th^ percentile (orange line), and 95^th^ percentile (purple line) number of diagnoses an individual classified as frail by the Hospital Frailty Risk Score had over time, stratified by age (i.e., 75, 85, 95) and sex.

glac210_suppl_Supplementary_Table_S1

glac210_suppl_Supplementary_Table_S2

glac210_suppl_Supplementary_Figure_S3

glac210_suppl_Supplementary_Figure_S4
