# Supplementary material for: Trends in Frailty Between 1990 and 2020 in Sweden Among 75-, 85-, and 95-Year-Old Women and Men: A Nationwide Study from Sweden
Source: J Gerontol A Biol Sci Med Sci. 2022 Oct 3;78(2):342–8. doi: 10.1093/gerona/glac210 (PMC9951059; doi:10.1093/gerona/glac210)
Supplement: glac210_suppl_Supplementary_Table_S2 [file glac210_suppl_supplementary_table_s2.pdf]

Supplementary Table 2. Swedish ICD 9 and 10 codes used to create the HFRS

ICD9	ICD10	ICD DESCRIPTION	WEIGHT
331A	F00	Dementia in Alzheimer's disease	7.1
342X	G81	Hemiplegia	4.4
331A	G30	Alzheimer's disease	4.0
438	I69	Sequelae of cerebrovascular disease	3.7
781X	R29	Other symptoms and signs involving the nervous and musculoskeletal systems	3.6
596X	N39	Other disorders of urinary system (includes urinary tract infection and urinary incontinence)	3.2
293	F05	Delirium, not induced by alcohol and other psychoactive substances	3.2
E888	W19	Unspecified fall	3.2
910W	S00	Superficial injury of head	3.2
599H	R31	Unspecified haematuria	3.0
041	B96	Other bacterial agents as the cause of diseases classified to other chapters	2.9
799X	R41	Other symptoms and signs involving cognitive functions and awareness	2.7
781C	R26	Abnormalities of gait and mobility	2.6
437X	I67	Other cerebrovascular diseases	2.6
780D	R56	Convulsions, not elsewhere classified	2.6
780A	R40	Somnolence, stupor and coma	2.5
996H	T83	Complications of genitourinary prosthetic devices, implants and grafts	2.4
854B	S06	Intracranial injury	2.4
812A	S42	Fracture of shoulder and upper arm	2.3
276	E87	Other disorders of fluid, electrolyte and acid-base balance	2.3
719	M25	Other joint disorders, not elsewhere classified	2.3
276	E86	Volume depletion	2.3
797	R54	Senility	2.2
V57W	Z51	Care involving use of rehabilitation procedures	2.1
294B	F03	Unspecified dementia	2.1
E885	W18	Other fall on same level	2.1
V63X	Z75	Problems related to medical facilities and other health care	2.0
290E	F01	Vascular dementia	2.0
916	S80	Superficial injury of lower leg	2.0
681	L03	Cellulitis	2.0
369	H54	Blindness and low vision	1.9
266C	E53	Deficiency of other B group vitamins	1.9
V62W	Z60	Problems related to social environment	1.8
332A	G20	Parkinson's disease	1.8
780C	R55	Syncope and collapse	1.8
807	S22	Fracture of rib(s), sternum and thoracic spine	1.8
564X	K59	Other functional intestinal disorders	1.8
584X	N17	Acute renal failure	1.8
707A	L89	Decubitus ulcer	1.7
V02	Z22	Carrier of infectious disease	1.7
041A	B95	Streptococcus and staphylococcus as the cause of diseases classified to other chapters	1.7
707B	L97	Ulcer of lower limb, not elsewhere classified	1.6
799W	R44	Other symptoms and signs involving general sensations and perceptions	1.6
532	K26	Duodenal ulcer	1.6
458X	I95	Hypotension	1.6

586	N19	Unspecified renal failure	1.6
038X	A41.9	Other septicaemia	1.6
V13	Z87	Personal history of other diseases and conditions	1.5
518W	J96	Respiratory failure, not elsewhere classified	1.5
V01X	X59	Exposure to unspecified factor	1.5
715	M19	Other arthrosis	1.5
345	G40	Epilepsy	1.5
733A	M81	Osteoporosis without pathological fracture	1.4
821A	S72	Fracture of femur	1.4
808W	S32	Fracture of lumbar spine and pelvis	1.4
251	E16	Other disorders of pancreatic internal secretion	1.4
794	R94	Abnormal results of function studies	1.4
585	N18	Chronic renal failure	1.4
788C	R33	Retention of urine	1.3
799X	R69	Unknown and unspecified causes of morbidity	1.3
593X	N28	Other disorders of kidney and ureter, not elsewhere classified	1.3
788D	R32	Unspecified urinary incontinence	1.2
331X	G31	Other degenerative diseases of nervous system, not elsewhere classified	1.2
136X	Y95	Nosocomial condition	1.2
959A	S09	Other and unspecified injuries of head	1.2
799C	R45	Symptoms and signs involving emotional state	1.2
435	G45	Transient cerebral ischaemic attacks and related syndromes	1.2
V60X	Z74	Problems related to care-provider dependency	1.1
729X	M79	Other soft tissue disorders, not elsewhere classified	1.1
E884	W06	Fall involving bed	1.1
873A	S01	Open wound of head	1.1
008W	A04	Other bacterial intestinal infections	1.1
009D	A09	Diarrhoea and gastroenteritis of presumed infectious origin	1.1
486	J18	Pneumonia, organism unspecified	1.1
507	J69	Pneumonitis due to solids and liquids	1.0
784F	R47	Speech disturbances, not elsewhere classified	1.0
268X	E55	Vitamin D deficiency	1.0
V44	Z93	Artificial opening status	1.0
785E	R02	Gangrene, not elsewhere classified	1.0
783X	R63	Symptoms and signs concerning food and fluid intake	0.9
389X	H91	Other hearing loss	0.9
E880	W10	Fall on and from stairs and steps	0.9
E885	W01	Fall on same level from slipping, tripping and stumbling	0.9
242X	E05	Thyrotoxicosis [hyperthyroidism]	0.9
737D	M41	Scoliosis	0.9
787C	R13	Dysphagia	0.8
V46W	Z99	Dependence on enabling machines and devices	0.8
V09	U82.0	Agent resistant to penicillin and related antibiotics	0.8
733A	M80	Osteoporosis with pathological fracture	0.8
536X	K92	Other diseases of digestive system	0.8
434X	I63	Cerebral Infarction	0.8
592B	N20	Calculus of kidney and ureter	0.7
291	F10	Mental and behavioural disorders due to use of alcohol	0.7
E879	Y84	Other medical procedures as the cause of abnormal reaction of the patient	0.7
785B	R00	Abnormalities of heart beat	0.7

519W	J22	Unspecified acute lower respiratory infection	0.7
V62	Z73	Problems related to life-management difficulty	0.6
790G	R79	Other abnormal findings of blood chemistry	0.6
V15X	Z91	Personal history of risk-factors, not elsewhere classified	0.5
881	S51	Open wound of forearm	0.5
296	F32	Depressive episode	0.5
724	M48.0	Spinal stenosis (secondary code only)	0.5
275	E83	Disorders of mineral metabolism	0.4
716F	M15	Polyarthrosis	0.4
285X	D64	Other anaemias	0.4
686X	L08	Other local infections of skin and subcutaneous tissue	0.4
787A	R11	Nausea and vomiting	0.3
558	K52	Other noninfective gastroenteritis and colitis	0.3
780G	R50	Fever of unknown origin	0.1
