# Supplementary figures and images for: Trends in Frailty Between 1990 and 2020 in Sweden Among 75-, 85-, and 95-Year-Old Women and Men: A Nationwide Study from Sweden
Source: J Gerontol A Biol Sci Med Sci. 2022 Oct 3;78(2):342–8. doi: 10.1093/gerona/glac210 (PMC9951059; doi:10.1093/gerona/glac210)

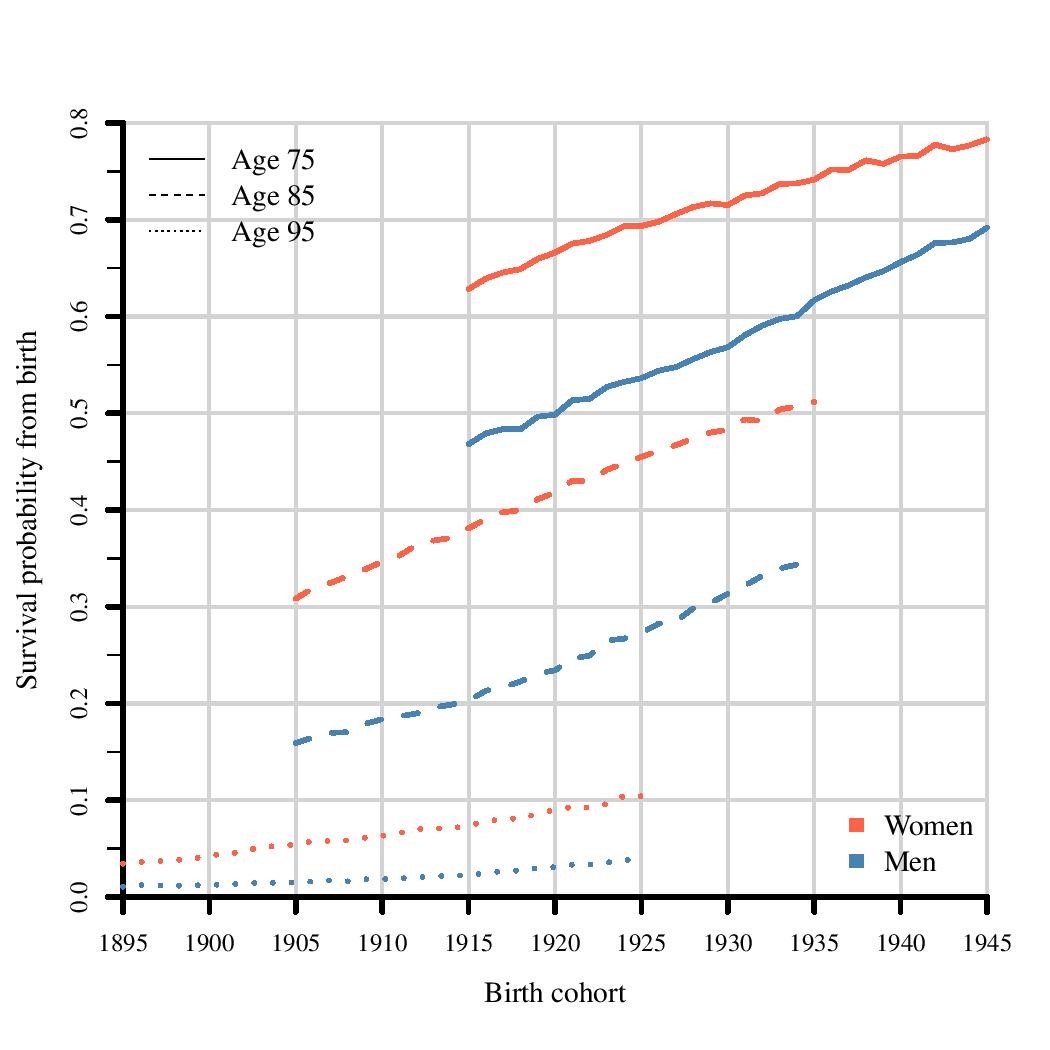

Supplement: glac210_suppl_Supplementary_Figure_S3 [file glac210_suppl_supplementary_figure_s3.jpeg]

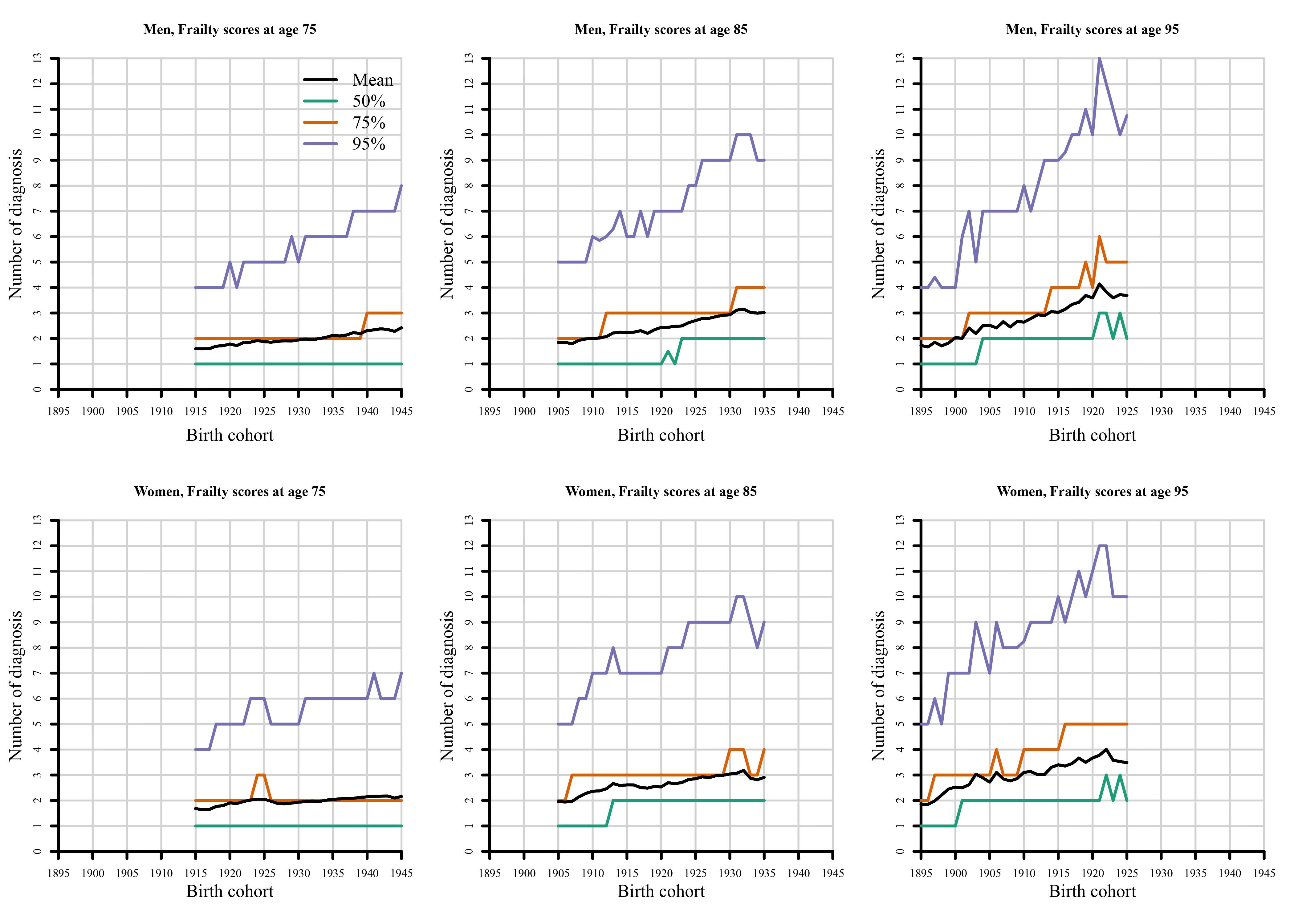

Supplement: glac210_suppl_Supplementary_Figure_S4 [file glac210_suppl_supplementary_figure_s4.jpeg]
